# Chondromyxoid Fibroma of the Calcaneus: A Rare Case Report

**DOI:** 10.7759/cureus.21950

**Published:** 2022-02-06

**Authors:** Koray Başdelioğlu, Mustafa İsmet Tatar, Burak Çağrı Aksu, Gokhan Meric

**Affiliations:** 1 Orthopedics and Traumatology, Faculty of Medicine, Yeditepe University, Istanbul, TUR; 2 Orthopedics and Traumatology, Denizli Tekden Hospital, Denizli, TUR

**Keywords:** bone tumor, bone graft, curettage, calcaneus, chondromyxoid fibroma

## Abstract

Chondromyxoid fibroma (CMF) of the calcaneus is extremely rare. We report a case of CMF of the calcaneus in a 34-year-old female. She had foot pain for one year and had increased pain for the last two months. The patient complained of limping due to the pain she felt. CMF of the calcaneus was treated with curettage and bone grafting. The patient was allowed to mobilize the very next day of surgery with weight bearing as much as she could tolerate. No recurrence was encountered during the 18-month follow-up of the patient. The patient could perform activities in her daily life painlessly. Carefully performed curettage and bone grafting is an effective treatment method in the treatment of CMF of the calcaneus. CMF in the calcaneus may not be as rare as it is thought, and should be considered in the differential diagnosis.

## Introduction

Chondromyxoid fibroma (CMF) is a very rare benign primary bone tumor [1]. CMFs are the rarest known cartilage tumor and make up less than 1% of all primary bone tumors [2-4]. CMF is seen in 75% of the long bones of the lower extremity [1,2]. Although some publications state that CMF is seen more frequently in males, there are also publications reporting that CMF is seen in females and males equally [3-5].

Although pathologic examination of CMF is difficult, CMF is distinguished from other bone tumors in histopathological examination with a plentiful combination of both myxoid and chondroid intercellular material with loculated areas of spindle-shaped or stellate cells and varying numbers of multinucleated giant cells of different sizes [6,7]. The presence of large pleomorphic cells in the histopathological evaluation may cause CMF to be confused with the diagnosis of chondrosarcoma, which is one of the closest differential diagnoses [7].

There are quite a few studies in the literature reporting CMF of the calcaneus [1,6,8-11]. In this case report, a 34-year-old female patient, who underwent curettage and spongiose grafting surgery with the diagnosis of CMF in the calcaneus, was presented.

## Case presentation

A 34-year-old female had pain in the left foot that had been going on for one year and had been exacerbated for the last two months. She complained of limping because of the pain she felt. There was no history of trauma. She did not have any disease and previous history of surgery. On examination of the patient, there was severe tenderness with palpation at the lateral of left foot calcaneocuboid joint level. Ankle and subtalar joint range of motion was in normal limits. In her neurological examination, there was no pathological finding.

**Figure 1:**
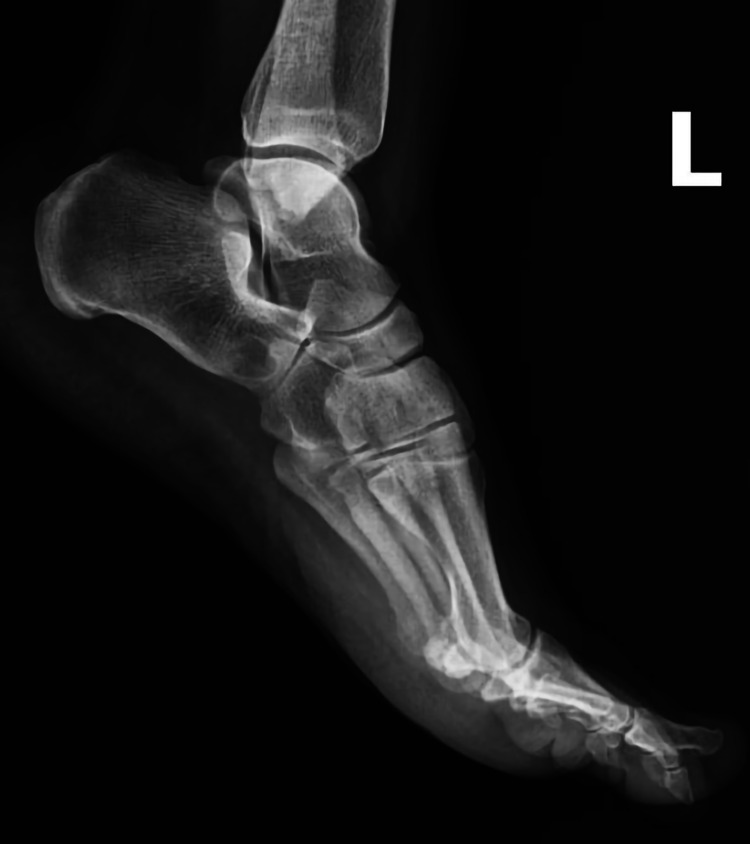
Preoperative lateral foot X-ray image

In the left foot X-ray images, it was observed that there was a sclerotic mass around the calcaneocuboid joint adjacent to the calcaneus (Figure 1).

**Figure 2:**
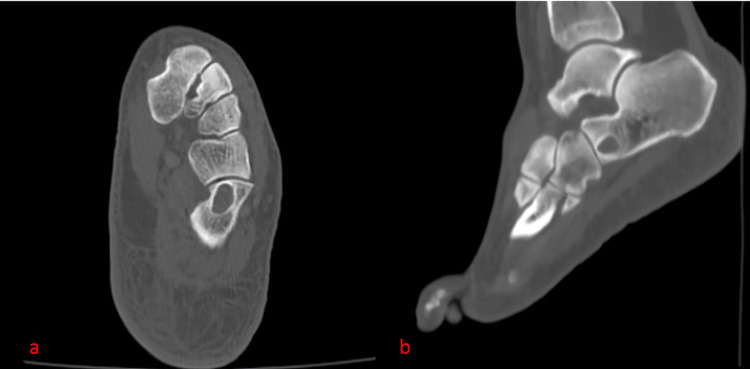
Mass surrounded by sclerotic rim adjacent to the calcaneocuboid joint in CT axial (a) and sagittal (b) images

A computerized tomography (CT) was performed on the left foot of the patient. The CT images showed a 13 x 6mm lesion with sclerotic rim in the calcaneus in the area adjacent to the calcaneocuboid joint (Figure 2).

**Figure 3:**
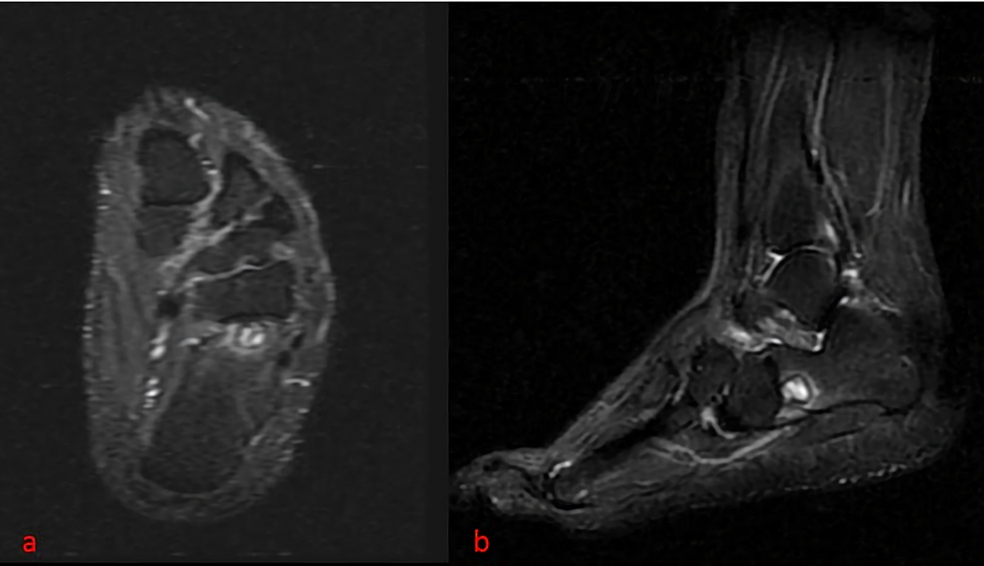
High signal intensity of the lesion in the MRI T2 axial series (a) and (b) sagittal series.

T2 weighted magnetic resonance imaging (MRI) of the lesion showed a high-intensity signal (Figure 3).

**Figure 4:**
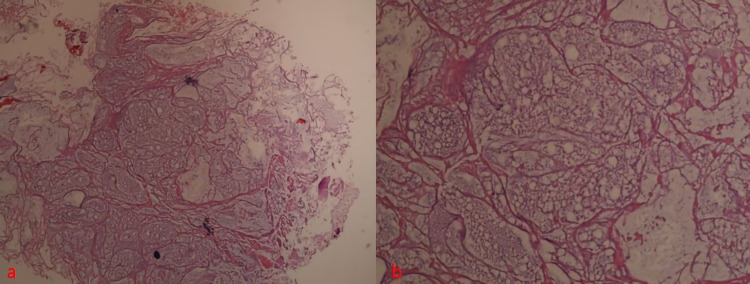
Histologic feature showing circumscribed nodular formation in H&E 50x (a) and fibrous bands and chondromyxomatous stroma in H&E 100x (b).

In the differential diagnosis of the patient, fibrous dysplasia, osteochondroma, osteoblastoma, giant cell tumor, aneurysmal bone cyst, unicameral bone cyst, nonossifying fibroma, intraosseous lipoma or ganglion, chondroblastoma, and CMF were considered. A bone biopsy was planned. A needle biopsy was performed and CMF was diagnosed as a result of examination of the samples (Figure 4). Surgery was planned.

Under spinal anesthesia, a skin incision was made immediately proximal to the calcaneocuboid joint in the lateral of the left foot. Then bone tissue was approached. In the localization to come over the mass in the calcaneus, a 2x1 cm bone window was opened by osteotome. The mass was excised through this bone window, curettage was done in the region of ​​the mass. The calcaneocuboid joint is preserved. The defect formed after curettage was filled with a spongiose bone graft. The bone removed as a window was replaced.

**Figure 5:**
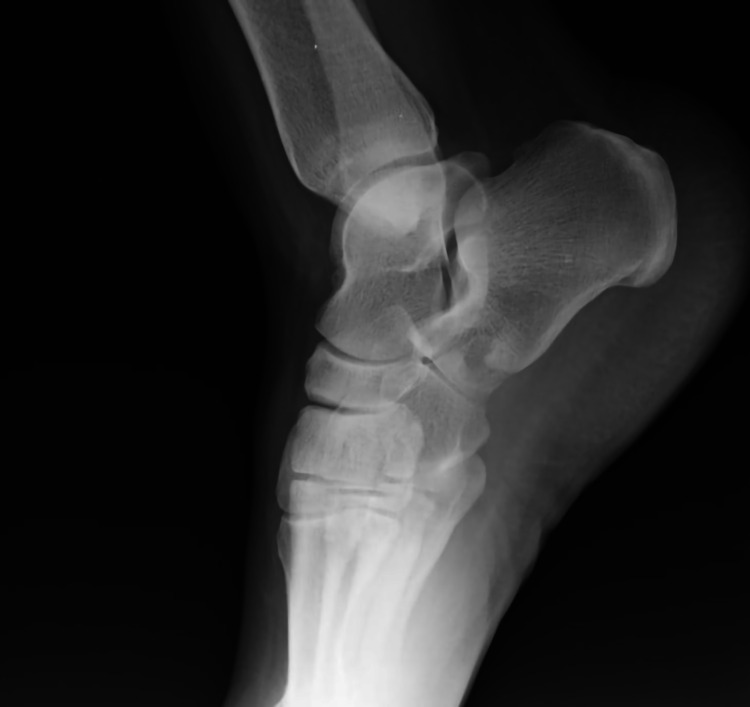
Lateral foot X-ray image taken at 18 months after surgery, in which normal trabecular pattern is seen in the graft region.

The patient was allowed to mobilize by weight bearing as much as she could tolerate the next day after surgery. The day after surgery, ankle and subtalar joint range of motion exercises were started. During the 18-month follow-up period, the patient had no symptoms and CMF recurrence. An X-ray image of the foot taken at the 18th month follow-up postoperatively is shown in Figure 5.

## Discussion

CMF is a very rare benign aggressive cartilaginous tumor of less than 0.5% of all bone tumors [6,12]. More than 75% of CMF occurs in long bones. The most common localizations are 53% proximal tibia, 27% distal femur and 20% fibula [13]. Calcaneus is a very rare localization for CMF [6]. Radiological and histological data should be carefully correlated in order to be able to diagnose this lesion, because 22-28% of cases are being misdiagnosed as being reported in the literature [7]. In this case report, the patient was diagnosed as CMF in the calcaneus in a region close to the calcaneocuboid joint neighborhood. The mass was excised by curettage and the occurred defect was filled with a spongiose bone graft.

Although the most common symptom in patients with CMF is chronic pain, the symptoms may not be seen in patients [7,13]. CMF may grow slowly and in the absence of symptoms, the diagnosis can be extended from months to years [9]. In the present case, the patient was symptomatic, although the lesion was relatively small. The patient had foot pain for about a year, which caused the limping.

Morphologically CMF has various differential diagnoses, however, one of the closest differential diagnoses is low-grade chondrosarcoma. It is of paramount importance to distinguish these tumors as treatment and prognosis differ [14].

The treatment of CMF is very challenging due to recurrence. There are cases in the literature treated with simple curettage, curettage and bone grafting, en bloc resection and even amputation [1,15]. There are quite a few studies in the literature reporting CMF cases in calcaneus [1,6,8-11]. Jamshidi et al. have the highest series with five cases of CMF patients of the calcaneus. They treated all their patients with curettage, high-speed burring and bone grafting. No cases of recurrence were observed as the result of their study [1]. Azami et al. treated a 22-year-old patient with CMF in the calcaneus with curettage and bone grafting [6]. Ebrahimzadeh and Dallouei treated a patient with CMF at the age of 10 with only curettage and did not encounter a recurrence in their three-year follow-up [8]. Roberts et al. treated two patients with CMF in the calcaneus by curettage and bone grafting [9]. Van Horn and Lemmens carried out knee amputation after the last recurrence in an 11-year-old patient treated with curettage four times with CMF diagnosis [10]. In this case report, the patient with CMF in the calcaneus was treated with curettage and bone grafting. No recurrence was encountered in the 18-month follow-up of the patient. The patient could perform activities in his daily life painlessly.

## Conclusions

Carefully performed curettage and bone grafting is an effective treatment method that reduces the patient's pain, increases the function and the quality of life in the treatment of CMF of the calcaneus. CMF in the calcaneus may not be as rare as it is thought, and should be considered in the differential diagnosis.
